# Effects of Ketamine on Levels of Inflammatory Cytokines IL-6, IL-1β, and TNF-α in the Hippocampus of Mice Following Acute or Chronic Administration

**DOI:** 10.3389/fphar.2017.00139

**Published:** 2017-03-20

**Authors:** Yanning Li, Ruipeng Shen, Gehua Wen, Runtao Ding, Ao Du, Jichuan Zhou, Zhibin Dong, Xinghua Ren, Hui Yao, Rui Zhao, Guohua Zhang, Yan Lu, Xu Wu

**Affiliations:** ^1^Department of Forensic Pathology, School of Forensic Medicine, China Medical University, ShenyangChina; ^2^Wujiang District Branch of Suzhou Public Security Bureau, SuzhouChina; ^3^Key Laboratory of Health Ministry in Congenital Malformation, The Affiliated Shengjing Hospital of China Medical University, ShenyangChina

**Keywords:** ketamine, intraperitoneal, acute, chronic, behavior, IL-6, IL-1β, TNF-α

## Abstract

Ketamine is an injectable anesthetic and recreational drug of abuse commonly used worldwide. Many experimental studies have shown that ketamine can impair cognitive function and induce psychotic states. Neuroinflammation has been suggested to play an important role in neurodegeneration. Meanwhile, ketamine has been shown to modulate the levels of inflammatory cytokines. We hypothesized that the effects of ketamine on the central nervous system are associated with inflammatory cytokines. Therefore, we set out to establish acute and chronic ketamine administration models in C57BL/6 mice, to evaluate spatial recognition memory and emotional response, to analyze the changes in the levels of the inflammatory cytokines interleukin-6 (IL-6), interleukin-1β (IL-1β), and tumor necrosis factor-α (TNF-α) in the mouse hippocampus, employing behavioral tests, Western blot, quantitative reverse transcriptase-polymerase chain reaction (qRT-PCR) and immunohistochemistry. Our results showed that ketamine at the dose of 60 mg/kg induced spatial recognition memory deficit and reduced anxiety-like behaviors in mice after chronic administration. Moreover, we found that ketamine increased the hippocampal levels of IL-6 and IL-1β after single, multiple and long-term administration in a dose-dependent manner. However, the expression level of TNF-α differed in the mouse hippocampus under different conditions. Single administration of ketamine increased the level of TNF-α, whereas multiple and long-term administration decreased it significantly. We considered that TNF-α expression could be controlled by a bi-directional regulatory pathway, which was associated with the dose and duration of ketamine administration. Our results suggest that the alterations in the levels of inflammatory cytokines IL-6, IL-1β, and TNF-α may be involved in the neurotoxicity of ketamine.

## Introduction

Ketamine, derived from phencyclidine classes (*N*-1-phenycyclohexypiperidine, PCP), acts as a non-competitive *N*-methyl-*D*-aspartate (NMDA) receptor antagonist (Martin and Lodge, 1985) by binding to the phencyclidine site (Orser et al., 1997), and has been commonly used as a type of clinical anesthetic since the 1960s (Haas and Harper, 1992). At low doses, ketamine can produce a psychedelic experience of incredible intensity (Strayer and Nelson, 2008). Because of these side reactions, it has become increasingly popular in recent years as a recreational drug (Lankenau et al., 2007). Experimental studies have shown that ketamine, either acute or chronic, can induce various cognitive impairments (Morgan et al., 2006; Chan et al., 2013; Duan et al., 2013). The acute application of ketamine gives rise to transient behavioral states in rats that mirror many symptoms of schizophrenia, such as motor disturbances and disturbed social behaviors (Becker et al., 2003; Razoux et al., 2007). With regard to the long-term effects of ketamine, one study found that mice exposed to a subanesthetic dose of ketamine (30 mg/kg) for 6 months showed a hyperphosphorylation of tau and apoptosis in the prefrontal and entorhinal cortex (Yeung et al., 2010). Another study performed with developing monkeys showed that prolonged exposure to ketamine increased cell death in brain areas (Zou et al., 2009). These findings suggested that acute and chronic ketamine administration may interfere with the central nervous system.

Neuroinflammation has been suggested to be involved in neurodegeneration. Inflammatory cytokines, including interleukin-6 (IL-6), interleukin-1β (IL-1β), and tumor necrosis factor-α (TNF-α), are cytokines involved in inflammation, which could be released quickly under pathological conditions, causing an inflammatory response in the central nervous system. IL-6, IL-1β, and TNF-α have been reported to be associated with learning and memory impairment (Reichenberg et al., 2001; Sparkman et al., 2006). Ketamine has the potential to modulate inflammation. A previous study showed that ketamine exerted an anti-inflammatory effect in the presence of inflammation, and recommended that it be used in the surgery of sepsis patients due to its anti-inflammatory effects (Takahashi et al., 2010). However, it was still unclear how the levels of IL-6, IL-1β, and TNF-α would change in the mouse hippocampus after short-term and long-term ketamine administration. We therefore set out to establish acute and chronic ketamine administration models in C57BL/6 mice at a subanesthetic dose, to determine whether mice exhibited changes in spatial memory and emotional response and to investigate the alterations in mouse hippocampal levels of IL-6, IL-1β, and TNF-α by applying the Y maze test, open field test, Western blot, qRT-PCR, and immunohistochemistry. We tried to determine the effects of short-term and long-term ketamine administration on the levels of IL-6, IL-1β, and TNF-α in mouse hippocampus, and to analyze the possible underlying relationship between behavioral performances and the alterations in the levels of IL-6, IL-1β, and TNF-α induced by ketamine, to provide preliminary evidence regarding the neurotoxicity of ketamine.

## Materials and Methods

### Animals

Sixty 2-month-old (for chronic experiment) and one hundred and twenty 3-month-old (for acute experiment) naive adult male C57BL/6 mice from Laboratory Animal Centre of China Medical University, weighing 22–28 g, were housed four per cage and maintained on a 12-h light/dark cycle (lights out at 5:00 PM). Mice had unlimited access to water and food in their home cages. All animal use procedures were in accordance with the Regulations of Experimental Animal Administration issued by the State Committee of Science and Technology of the People’s Republic of China and the Guidelines of the Care and Use of Laboratory Animals of China Medical University. The experimental techniques were approved by the Institutional Animal Care and Use Committee of China Medical University. All efforts were made to minimize the number of animals used and to reduce their suffering.

### Ketamine Administration

Ketamine hydrochloride (Fujian Gutian Pharmaceutical Co., Ltd, Gutian, Fujian, China), was dissolved in physiological saline.

In the acute experiment, we gave mice a single intraperitoneal (i.p.) injection of ketamine or multiple (six times) consecutive i.p. injections of ketamine at 1-h intervals, based on the methods described in previous studies (Huang et al., 2015, 2016), with modifications. For both single and multiple administrations, mice were randomly assigned to five groups, with 12 mice per group: saline-treated group, 10 mg/kg ketamine group, 20 mg/kg ketamine group, 40 mg/kg ketamine group, and 80 mg/kg ketamine group. Animals received i.p. injection of ketamine or equal volume of physiological saline according to group, for a single time injection or six times consecutive injections at 1-h intervals. After behavioral tests, all mice of each group were used for proteomic and transcriptomic analysis.

In the chronic experiment, we gave mice a 6-month course of daily i.p. injection of ketamine, as described in previous studies (Yeung et al., 2010; Sun et al., 2011). Mice were randomly assigned to three groups, with 20 mice per group: saline-treated group, 30 mg/kg ketamine group and 60 mg/kg ketamine group. Animals received i.p. injection of ketamine or equal volume of physiological saline according to group for 6 months. The body weights of mice were recorded every week for adjustment of ketamine administration. After behavioral tests, 10 mice of each group were randomly selected for immunohistochemical staining, and the other 10 mice used for proteomic and transcriptomic analysis.

We used subanesthetic doses of 30 and 60 mg/kg for chronic administration and 10, 20, 40, and 80 mg/kg for acute administration, which were based on the doses reported in previous studies (Hunt et al., 2006; Enomoto and Floresco, 2009; Wang et al., 2011, 2014), with modifications. All mice were sacrificed by cervical dislocation 6 h after the last i.p. injection.

### Behavioral Tests

All behavioral tests were performed 3 h after the last i.p. injection. All mice were in a normal state before and during the behavior acquisition. All procedures were performed during the light period. Mice that exhibited passivity were excluded from analysis.

### Open Field Test

We used the open field test to evaluate anxiety-like response after ketamine administration, as previously described (Akillioglu et al., 2012). The open field apparatus was a four-walled black plastic box that measured 40 cm × 40 cm × 30 cm (length/width/height) with a white bottom and no top. The squares adjacent to the wall were designated as “peripheral,” while the others were designated as “central” (23 cm × 23 cm). Peripheral fields were safe and protected, whereas the central fields were unprotected in the open field apparatus. For the open field test, mice were placed in a corner of the apparatus. All environments were thoroughly cleaned with 70% ethanol between trials. Behavioral performances were recorded for 10 min using a video camera. SMART^TM^ tracking software program (San Diego Instruments, San Diego, CA, USA) was used to calculate the time spent in the center of the open field.

### Y Maze Test

We used the Y maze test to measure spatial recognition memory, as previously described (Dellu et al., 2000; Ma et al., 2007). The Y maze apparatus consisted of three arms with an angle of 120° between each two arms. Each arm of the maze was 30 cm long, 15 cm high, and 6 cm wide, and converged at an equal angle. The three identical arms were randomly designated as follows: initial arm, in which the mouse started to explore; novel arm, which was blocked during the first trial, but open during the second trial; and familiar arm. Visual cues were placed on the walls of the mazes. The Y maze test consisted of two trials separated by 2-h intervals to assess spatial recognition memory. The first trial (training) lasted 8 min and allowed mice to explore only the initial arm and familiar arm of the maze, with the novel arm being blocked. After 2-h intervals, the second trial was conducted for 2 min, during which all three arms were accessible. All environments were thoroughly cleaned with 70% ethanol between trials. This test is based on the interest of mice for novelty, hence they explore preferentially unknown territories. Behavioral performances were recorded using a video camera. SMART^TM^ tracking software program (San Diego Instruments, San Diego, CA, USA) was used to calculate the time spent in the novel arm.

### Western Blot Assay

Western blot analysis was used to determine the protein levels of IL-6, IL-1β, and TNF-α as previously described (Lu et al., 2010), with modifications. Briefly, mice were sacrificed by cervical dislocation, and the hippocampus was dissected out. Hippocampal cytosol proteins were extracted using the Membrane and Cytosol Protein Extraction Kit (P0033, Beyotime, Shanghai, China) and quantified for total protein with the BCA Protein Assay Kit (P0012, Beyotime, Shanghai, China). Next, 20 μg of protein of each lysate were separated by SDS-PAGE and the bands transferred to PVDF membranes. After being blocked with 4% bovine serum albumin in Tris-buffered saline-Tween-20 at room temperature for 2 h, the membranes were incubated, respectively, with anti-IL-6 (1:2000, 21865-1-AP, Proteintech), anti-IL-1β (1:2000, 16765-1-AP, Proteintech), anti-TNF-α (1:2000, ab9739, Abcam), or anti-β-actin (1:5000, ab8226, Abcam) overnight at 4°C. After washing, the membranes were incubated with appropriate horseradish peroxidase-conjugated secondary antibodies (1:5000, ZSGB-BIO, Beijing, China) at room temperature for 2 h. The blots were visualized with luminal reagent, and images were taken and analyzed by Electrophoresis Gel Imaging Analysis System (Tanon 5500, Shanghai, China).

### Immunohistochemistry Assay

Mice were fully anesthetized with chloral hydrate (400 mg/kg, i.p.) and then transcardially perfused with phosphate-buffered saline (PBS) (pH 7.4) followed by 4% paraformaldehyde dissolved in 0.1 M phosphate buffer (pH 7.4). The brain was then removed from the skull and fixed at 4°C overnight in 4% paraformaldehyde. The hippocampus was embedded with paraffin and then sectioned coronally at a thickness of 5 μm. For immunohistochemical staining, deparaffinized sections were heated for 5 min in 0.01 M sodium citrate buffer (pH 6.0) for antigen retrieval. Endogenous peroxide activity was quenched with 3% hydrogen peroxide, and non-specific binding was blocked with non-immune serum. The sections were then incubated, respectively, with anti- IL-6 (1:300, 21865-1-AP, Proteintech), anti-IL-1β (1:300, 16765-1-AP, Proteintech), anti-TNF-α (1:300, ab9739, Abcam) at 4°C overnight, and followed by incubation with appropriate biotinylated secondary antibody in SPlink Detection Kit (ZSGB-BIO, Beijing, China) for 1 h at 37°C. The immunostaining positive cells in the mouse hippocampus were detected using a light microscope (Olympus BX 41, Japan). Images were captured. The relative expression levels of IL-6, IL-1β, and TNF-α were quantified by the mean optical density (MOD) value. Measurements were performed as follows: integrated optical density (IOD) and total stained area (S) in each image were recorded using the Image-Pro Plus 6.0 software (Media Cybernetics, Rockville, MD, USA). MOD = IOD/S, equivalent to the intensity of stain in all positive cells.

### Quantitative Reverse Transcriptase-Polymerase Chain Reaction (qRT-PCR)

The qRT-PCR method was performed as previously described (Wu et al., 2012) with modifications, not following the MIQE guidelines. Briefly, total RNA was isolated with TRIzol according to the manufacturer’s instructions. RNA was reversely transcribed into complementary deoxyribonucleic acid (cDNA) using PrimeScript^TM^ RT Reagent Kit (RR037A, Takara Biotechnology, Shiga, Japan). RNA concentration was determined using a NanoDrop ND-1000 Spectrophotometer (Thermo Fisher Scientific, Wilmington, DE, USA). Real-time PCR amplification was carried out by Applied Biosystems 7500 Real-time PCR System using the SYBR^®^ PrimeScript^TM^ RT-PCR Kit (RR820A, Takara Biotechnology, Shiga, Japan). IL-6, IL-1β, and TNF-α mRNA levels were determined and standardized with mouse glyceraldehyde 3-phosphate dehydrogenase (GAPDH) as internal control. No significant changes in GAPDH mRNA levels were observed. Primer sequences were as follows: IL-6, forward: 5′- CAAAGCCAGAGTCCTTCAGAG -3′, reverse: 5′- GTCCTTAGCCACTCCTTCTG -3′; IL-1β, forward: 5′- ACGGACCCCAAAAGATGAAG -3′, reverse: 5′- TTCTCCACAGCCACAATGAG -3′; TNF-α, forward: 5′- CTTCTGTCTACTGAACTTCGGG -3′, reverse: 5′- CAGGCTTGTCACTCGAATTTTG -3′; and GAPDH, forward: 5′- CTTTGTCAAGCTCATTTCCTGG -3′, reverse: 5′- TCTTGCTCAGTGTCCTTGC -3′. To exclude any potential contamination, negative controls were also performed with dH_2_O instead of cDNA during each run, and no amplification product was detected. The real-time PCR procedure was repeated at least three times for each sample. Relative quantification method was used to calculate the difference in gene expression (Livak and Schmittgen, 2001). The data were presented as fold changes in target gene expression normalized to GAPDH.

### Statistical Analysis

One-way analysis of variance (ANOVA) followed by the least significant difference (LSD) *t*-test was used to compare differences between groups. These statistical analyses were conducted by Statistical Product for Social Sciences (SPSS version 13.0). *p*-values less than 0.05 (* or #) and 0.01 (** or ##) were considered statistically significant.

## Results

### Open Field Test

**FIGURE 1:**
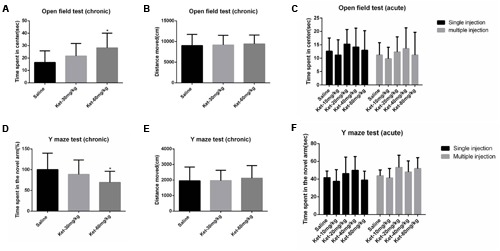
**Effects of acute and chronic intraperitoneal injection of ketamine on anxiety-like behaviors in the open field test and spatial recognition memory in the Y maze test. (A)** Time spent in center of the open field (chronic). **(B)** Total distance moved in the open field (chronic). **(C)** Acute effects of ketamine in the open field test. **(D)** The percentage of time spent in the novel arm with respect to the total duration of visits in the three arms of the Y maze (chronic). **(E)** Total distance moved in the Y maze (chronic). **(F)** Acute effects of ketamine in the Y maze test. Data are expressed as mean ± SD, *n* = 12 for each group. Statistical analysis used ANOVA to compare the difference between groups (*p < 0.05).

In the chronic experiment, mice that received 6 months of daily administration of 60 mg/kg ketamine significantly spent more time in the center of the open field compared to the saline-treated group [**Figure 1A**; *F*_(2,33)_ = 3.973, *p* = 0.028], but not the 30 mg/kg group. No significant differences were observed between 60 and 30 mg/kg ketamine groups after long-term administration. Total distance moved in the open field was not significantly affected by chronic ketamine administration [**Figure 1B**; *F*_(2,33)_ = 0.104, *p* = 0.902]. In the acute experiment, our results showed that ketamine at all doses (10, 20, 40, and 80 mg/kg), whether with single [**Figure 1C**; *F*_(4,55)_ = 1.025, *p* = 0.403] or multiple [**Figure 1C**; *F*_(4,55)_ = 0.836, *p* = 0.508] administration, did not induce any significant differences in the time spent in the center of the open field compared to the saline-treated group. The data from the open field test indicated that ketamine treatment at 60 mg/kg for 6 months reduced anxiety-like behaviors in mice, whereas acute administration had no effect.

### Y Maze Test

We used the Y maze test to determine the effects of ketamine administration on spatial recognition memory. Results showed that mice that received 6 months of daily ketamine administration at a dose of 60 mg/kg exhibited a significant reduction in the percentage of time spent in the novel arm with respect to the total duration of visits in the three arms of Y maze compared to the saline-treated group [**Figure 1D**; *F*_(2,33)_ = 3.616, *p* = 0.038] but not the 30 mg/kg group. No significant differences were observed between the 60 and 30 mg/kg ketamine groups after long-term ketamine administration. Total distance moved in the Y maze was not significantly affected by chronic ketamine administration [**Figure 1E**; *F*_(2,33)_ = 0.129, *p* = 0.879]. Similar to the open field test results, no significant differences were observed between the saline-treated group and each ketamine-treated group (10, 20, 40, and 80 mg/kg) after acute ketamine administration, whether with single [**Figure 1F**; *F*_(4,55)_ = 1.892, *p* = 0.125] or multiple [**Figure 1F**; *F*_(4,55)_ = 2.021, *p* = 0.104] administration. The Y maze test results indicated a spatial recognition memory deficit with the 6-month administration paradigm for 60 mg/kg ketamine, whereas acute ketamine administration had no effect.

### Single Administration of Ketamine Increased the Protein Levels of IL-6, IL-1β, and TNF-α in Mouse Hippocampus 6 h after Injection

**FIGURE 2:**
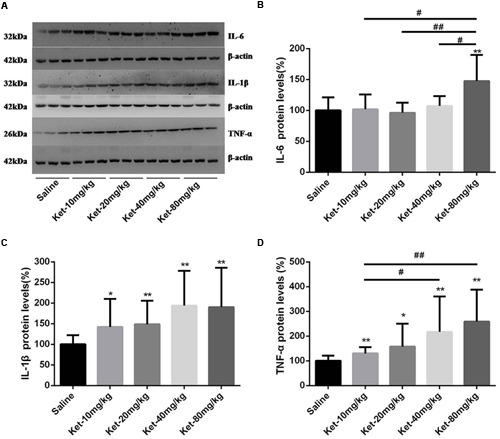
**Single intraperitoneal injection of ketamine increased the protein levels of IL-6, IL-1β, and TNF-α in mouse hippocampus as compared with saline-treated group 6 h after administration. (A)** Effects of single intraperitoneal injection of ketamine on IL-6, IL-1β, and TNF-α levels. **(B)** Quantification of the Western blot shows that only the single intraperitoneal injection of 80 mg/kg ketamine significantly increased IL-6 level normalized to β-actin as compared with saline-treated group. **(C)** Quantification of the Western blot shows that all doses of single intraperitoneal injection of ketamine significantly increased IL-1β level normalized to β-actin as compared with saline-treated group. **(D)** Quantification of the Western blot shows that all doses of single intraperitoneal injection of ketamine significantly increased TNF-α level normalized to β-actin as compared with saline-treated group. Data are expressed as mean ± SD, *n* = 12 for each group. Statistical analysis used ANOVA to compare the difference between saline-treated group and each ketamine-treated group (*p < 0.05; **p < 0.01) and the differences between ketamine-treated groups (#*p* < 0.05; ##*p* < 0.01).

Inflammatory cytokines, including IL-6, IL-1β, and TNF-α, have been reported to be associated with learning and memory impairment. Therefore, we determined the hippocampal cytosol protein levels of IL-6, IL-1β, and TNF-α. Our Western blot results revealed that only the single administration of 80 mg/kg ketamine significantly increased IL-6 level normalized to β-actin as compared with the saline-treated group [**Figures 2A,B**; *F*_(4,55)_ = 9.17, *p* < 0.001]. Lower doses of ketamine (10, 20, and 40 mg/kg) did not induce an evident increase in IL-6 level. Moreover, the 80 mg/kg ketamine group displayed a markedly higher level of IL-6 compared to the other ketamine administration groups (10, 20, and 40 mg/kg) [**Figure 2B**; #*p* < 0.05, ##*p* < 0.01]. Somewhat differently, all ketamine doses (10, 20, 40, and 80 mg/kg) produced a significant increase in IL-1β [**Figures 2A,C**; *F*_(4,55)_ = 3.728, *p* = 0.009] and TNF-α levels [**Figures 2A,D**; *F*_(4,55)_ = 4.475, *p* = 0.003]. Meanwhile, the 40 and 80 mg/kg ketamine groups exhibited a markedly higher level of TNF-α compared to the 10 mg/kg ketamine group [**Figure 2D**; #*p* < 0.05, ##*p* < 0.01]. These results indicated that single administration of ketamine increased the protein levels of IL-6 (only at the high dose of 80 mg/kg), IL-1β and TNF-α in mouse hippocampus, but that low doses of ketamine (10, 20, and 40 mg/kg) had no effect on the level of IL-6.

### Multiple (Six Times) Consecutive Administration of Ketamine at 1-h Intervals Increased the Protein Levels of IL-6 and IL-1β, but Decreased the Protein Levels of TNF-α in Mouse Hippocampus 6 h after the Last Injection

**FIGURE 3:**
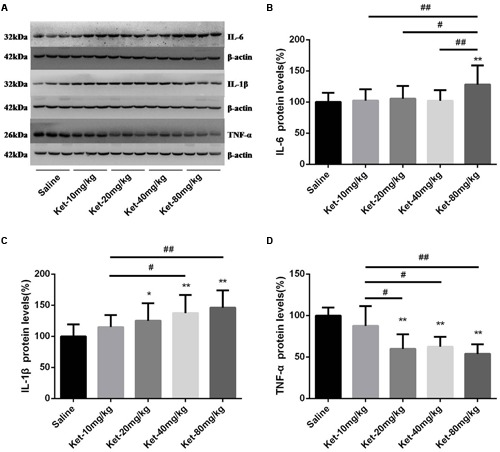
**Multiple (six times) consecutive intraperitoneal injection of ketamine at 1-h intervals increased the protein levels of IL-6 and IL-1β but decreased the protein levels of TNF-α in mouse hippocampus as compared with saline-treated group 6 h after the last injection. (A)** Effects of six times consecutive intraperitoneal injection of ketamine at 1-h intervals on IL-6, IL-1β, and TNF-α levels. **(B)** Quantification of Western blot shows that six times consecutive intraperitoneal injection of ketamine at 1-h intervals at only 80 mg/kg significantly increased IL-6 level normalized to β-actin as compared with saline-treated group. **(C)** Quantification of Western blot shows that six times consecutive intraperitoneal injection of ketamine at 1-h intervals at the doses of 20, 40, and 80 mg/kg significantly increased IL-1β level normalized to β-actin as compared with saline-treated group. **(D)** Quantification of Western blot shows that the six times consecutive intraperitoneal injection of ketamine at 1-h intervals at the doses of 20, 40, and 80 mg/kg significantly decreased TNF-α level normalized to β-actin as compared with saline-treated group. Data are expressed as mean ± SD, *n* = 12 for each group. Statistical analysis used ANOVA to compare the difference between saline-treated group and each ketamine-treated group (*p < 0.05; **p < 0.01) and the differences between ketamine-treated groups (#*p* < 0.05; ##*p* < 0.01).

We further investigated hippocampal cytosol protein levels of IL-6, IL-1β, and TNF-α after six times consecutive administration. Similarly, Our Western blot results revealed that only 80 mg/kg ketamine significantly increased IL-6 level as compared with the saline-treated group [**Figures 3A,B**; *F*_(4,55)_ = 3.737, *p* = 0.009]. Lower doses of ketamine (10, 20, and 40 mg/kg) did not induce a significant increase in IL-6 level. Moreover, the 80 mg/kg ketamine group displayed a markedly higher IL-6 level compared to the 10, 20, and 40 mg/kg ketamine groups [**Figure 3B**; #*p* < 0.05, ##*p* < 0.01]. The six times consecutive administration of ketamine at 1-h intervals at doses of 20, 40, and 80 mg/kg significantly increased IL-1β level as compared with the saline-treated group but not the 10 mg/kg ketamine group [**Figures 3A,C**; *F*_(4,55)_ = 6.347, *p* < 0.001]. However, it is noteworthy that TNF-α level significantly decreased after the six times consecutive administration of ketamine at 1-h intervals at doses of 20, 40, and 80 mg/kg (not 10 mg/kg) as compared with saline-treated group [**Figures 3A,D**; *F*_(4,55)_ = 23.77, *p* < 0.001]. Moreover, the 20, 40, and 80 mg/kg ketamine groups displayed a markedly lower level of TNF-α compared to the 10 mg/kg ketamine group [**Figure 3D**; #*p* < 0.05, ##*p* < 0.01]. These findings suggest that multiple (six times) administration of ketamine increased the protein level of IL-6 (only at the high dose of 80 mg/kg) and IL-1β (at doses of 20, 40, and 80 mg/kg) but decreased the protein level of TNF-α (at doses of 20, 40, and 80 mg/kg) in mouse hippocampus.

### Effects of Chronic (6 Months Daily) Administration of 30 and 60 mg/kg Ketamine on the Protein Levels and Immunoreactivity Levels of IL-6, IL-1β, and TNF-α

**FIGURE 4:**
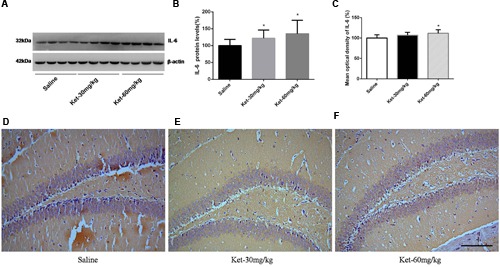
**Chronic (6 months daily) intraperitoneal injection of ketamine at 30 and 60 mg/kg increased the protein level and the immunostaining level of IL-6. (A)** Six months intraperitoneal injection of ketamine increased the protein level of IL-6. **(B)** Quantification of Western blot shows that 6 months intraperitoneal injection of ketamine significantly increased IL-6 level normalized to β-actin as compared with saline-treated group. **(C)** Quantification of the immunohistochemistry image shows that the ketamine administration at the 60 mg/kg significantly increased the IL-6 immunostaining level in mouse hippocampus. **(D–F)** Immunohistochemistry studies show that the ketamine administration increased the IL-6 immunostaining level in mouse hippocampus. Scale bar = 100 μm. Data are expressed as mean ± SD, *n* = 10 of each group for proteomic analysis and *n* = 6 of each group for immunohistochemical staining. Statistical analysis used ANOVA to compare the difference between saline-treated group and each ketamine-treated group (*p < 0.05).

**FIGURE 5:**
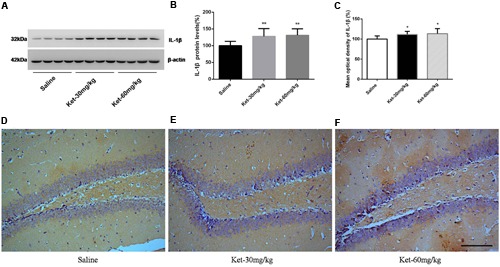
**Chronic (6 months daily) intraperitoneal injection of ketamine at 30 and 60 mg/kg increased the protein level and the immunostaining level of IL-1β. (A)** Six months intraperitoneal injection of ketamine increased the protein level of IL-1β. **(B)** Quantification of Western blot shows that 6 months intraperitoneal injection of ketamine significantly increased IL-1β level normalized to β-actin as compared with saline-treated group. **(C)** Quantification of the immunohistochemistry image shows that the ketamine administration at 30 and 60 mg/kg significantly increased the IL-1β immunostaining level in mouse hippocampus. **(D–F)** Immunohistochemistry studies show that the ketamine administration increased the IL-1β immunostaining level in mouse hippocampus. Scale bar = 100 μm. Data are expressed as mean ± SD, *n* = 10 of each group for proteomic analysis and *n* = 6 of each group for immunohistochemical staining. Statistical analysis used ANOVA to compare the difference between saline-treated group and each ketamine-treated group (*p < 0.05; **p < 0.01).

**FIGURE 6:**
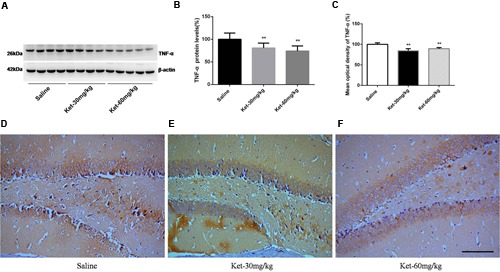
**Chronic (6 months daily) intraperitoneal injection of ketamine at 30 and 60 mg/kg decreased the protein level and the immunostaining level of TNF-α. (A)** Six months intraperitoneal injection of ketamine decreased the protein level of TNF-α. **(B)** Quantification of Western blot shows that 6 months intraperitoneal injection of ketamine significantly decreased TNF-α level normalized to β-actin as compared with saline-treated group. **(C)** Quantification of the immunohistochemistry image shows that the ketamine administration at 30 and 60 mg/kg significantly decreased the TNF-α immunostaining level in mouse hippocampus. **(D–F)** Immunohistochemistry studies show that the ketamine administration decreased the TNF-α immunostaining level in mouse hippocampus. Scale bar = 100 μm. Data are expressed as mean ± SD, *n* = 10 of each group for proteomic analysis and *n* = 6 of each group for immunohistochemical staining. Statistical analysis used ANOVA to compare the difference between saline-treated group and each ketamine-treated group (**p < 0.01).

The chronic effects of ketamine on inflammatory cytokines were investigated, where we determined the hippocampal immunoreactivity levels and the hippocampal cytosol protein levels of IL-6, IL-1β, and TNF-α after long-term (6 months daily) administration of ketamine at the doses of 30 and 60 mg/kg. Like the results for multiple (six times) consecutive injections, 6 months daily ketamine administration of 30 and 60 mg/kg increased the protein level [**Figures 4A,B**; *F*_(2,27)_ = 3.948, *p* = 0.031] and immunostaining level of IL-6 (at dose of 60 mg/kg) [**Figures 4C–F**; *F*_(2,15)_ = 4.313, *p* = 0.033], the protein level [**Figures 5A,B**; *F*_(2,27)_ = 6.782, *p* = 0.004] and immunostaining level of IL-1β [**Figures 5C–F**; *F*_(2,15)_ = 4.732, *p* = 0.026] but decreased the protein level of TNF-α [**Figures 6A,B**; *F*_(2,27)_ = 9.012, *p* = 0.001] and immunostaining level of TNF-α [**Figures 6C–F**; *F*_(2,15)_ = 19.1, *p* < 0.001] in mouse hippocampus. No significant differences were observed between the 60 and 30 mg/kg ketamine groups after long-term administration. These results suggest that ketamine may increase the levels of IL-6 and IL-1β but may decrease the level of TNF-α in mouse hippocampus after long-term administration.

### Effects of Acute and Chronic Ketamine Administration on the mRNA Levels of IL-6, IL-1β, and TNF-α

**FIGURE 7:**
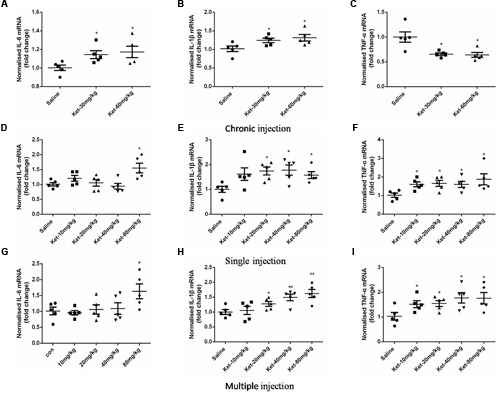
**Effects of acute and chronic ketamine administration on the mRNA levels of IL-6, IL-1β, and TNF-α in mouse hippocampus. (A–C)** The results of qRT-PCR show that 6 months intraperitoneal injection of ketamine increased the mRNA levels of IL-6 and IL-1β, but decreased the mRNA level of TNF-α. **(D–F)** The results of qRT-PCR show that single intraperitoneal injection of ketamine increased the mRNA levels of IL-6, IL-1β, and TNF-α. **(G–I)** The results of qRT-PCR show that multiple intraperitoneal injection of ketamine increased the mRNA levels of IL-6, IL-1β, and TNF-α. Data are expressed as mean ± SEM, *n* = 5 for each group. Statistical analysis used ANOVA to compare the difference between saline-treated group and each ketamine-treated group (*p < 0.05; **p < 0.01).

The changes in IL-6, IL-1β, and TNF-α levels after ketamine administration could have been due to the changes in synthesis or degradation. We therefore assessed the effects of ketamine administration on the mRNA levels of IL-6, IL-1β, and TNF-α. Our results showed that 6 months daily administration of 30 and 60 mg/kg ketamine increased the mRNA levels of IL-6 [**Figure 7A**; *F*_(2,12)_ = 3.92, *p* = 0.048] and IL-1β [**Figure 7B**; *F*_(2,12)_ = 4.199, *p* = 0.042], but decreased the mRNA levels of TNF-α [**Figure 7C**; *F*_(2,12)_ = 8.984, *p* = 0.004] as compared with the saline-treated group, using GAPDH as an internal control. In acute experiment, ketamine significantly increased the mRNA levels of IL-6 (at dose of 80 mg/kg) [**Figure 7D**; *F*_(4,20)_ = 4.895, *p* = 0.007], IL-1β (at doses of 20, 40, and 80 mg/kg) [**Figure 7E**; *F*_(4,20)_ = 2.944, *p* = 0.046] and TNF-α (at all doses) [**Figure 7F**; *F*_(4,20)_ = 2.907, *p* = 0.047] after single administration. Similarly, ketamine also significantly increased the mRNA levels of IL-6 (at dose of 80 mg/kg) [**Figure 7G**; *F*_(4,20)_ = 2.932, *p* = 0.046], IL-1β (at doses of 20, 40, and 80 mg/kg) [**Figure 7H**; *F*_(4,20)_ = 5.809, *p* = 0.003] and TNF-α (at all doses) [**Figure 7I**; *F*_(4,20)_ = 2.901, *p* = 0.048] after multiple administration. These results revealed that ketamine may affect IL-6, IL-1β, and TNF-α expression levels in mouse hippocampus at the transcription level.

## Discussion

In the present study, our findings indicate that hippocampal inflammatory cytokines IL-6, IL-1β, and TNF-α may be involved in the subanesthetic ketamine-induced behavioral changes in mice.

The Y maze task is a specific and sensitive test of spatial recognition memory in rodents (Dellu et al., 2000). A large number of studies have shown that long-term ketamine administration markedly induces learning and memory impairment in humans (Morgan et al., 2004; Chan et al., 2013) and mice (Tan et al., 2011). In our study, we used the Y maze test to measure spatial recognition memory, and found that only the mice that received chronic ketamine administration at 60 mg/kg (not 30 mg/kg) exhibited spatial memory deficit. This was supported by a study in which mice exposed to 30 mg/kg ketamine for 6 months showed no apparent deficit in learning and memory (Sun et al., 2011), and another study in which rats exhibited learning and memory impairment when exposed to ketamine at 80 mg/kg but not at a lower dose of 30 mg/kg in a 7 days administration paradigm (Wang et al., 2014). Previous works by our research group showed that a higher dose of ketamine (60 mg/kg) resulted in significant spatial learning and memory impairment after 6 months administration, while lower dose of ketamine (30 mg/kg) did not induce any apparent deficits in Morris water maze tests and radial arm maze test (Ding et al., 2016). These results indicate that cognitive function impairments after chronic ketamine administration are dose-dependent in mice. A previous study reported that a single i.p. injection of ketamine at subanesthetic and anesthetic doses did not impair recognition memory or reference memory and did not cause neurodegeneration in adult mice (Ribeiro et al., 2013). Similarly, we did not observe a significant difference between the saline-treated group and each ketamine-treated group (10, 20, 40, or 80 mg/kg) after acute administration, whether with single or multiple administration. We can conclude that acute ketamine administration has no effect on spatial recognition memory in mice.

In the open field test, our results showed that chronic ketamine administration at high dose had a significant anxiolytic effect. Mice displayed reduced anxiety-like behaviors after long-term administration at the dose of 60 mg/kg, while low dose ketamine (30 mg/kg) had no effect. Moreover, we did not observe significant differences after acute ketamine administration, whether with single or multiple administration. The results of previous studies investigating the anxiety-related acute effects of ketamine were completely different, with reports of anxiolytic (Imre et al., 2006; Engin et al., 2009), anxiogenic (da Silva et al., 2010; Akillioglu et al., 2012), and null (Sobota et al., 2015) results. Some of the conclusions were inconsistent with our results. The reason may be due to the fact that we performed the behavioral tests 3 h after the last injection of ketamine or saline, whereas the above-mentioned studies involved a much shorter period (15–90 min) after ketamine injection. Ketamine has a short half-life, and its metabolites are quickly cleared by urinary excretion (Sinner and Graf, 2008). In our study, we aimed to determine the stable and persistent effects of ketamine on the mouse central nervous system rather than its immediate effect. The acute application of ketamine may just give rise to transient behavioral states, so behavioral tests performed a long time (such as 3 h) after acute administration of ketamine may not show effects on emotional response and cognitive function in mice.

Neuroinflammation has been suggested to play an important role in neurodegeneration (Heneka and O’Banion, 2007; Cameron and Landreth, 2010), and to cause cognitive impairment both in humans (Hoogman et al., 2007) and animals (Langdon et al., 2008). There is extensive evidence that IL-6, as a proinflammatory cytokine, may affect brain function and may be involved in pathological neurodegenerative disorders. Studies have reported a positive association between plasma IL-6 level and impairment in a wide range of cognitive domains in humans (Gimeno et al., 2008; Simpson et al., 2013; Heringa et al., 2014). Meanwhile, IL-6 overexpression in the brain of transgenic mice has been shown to cause severe neurological disease (Heyser et al., 1997). IL-6 knockout mice have been found to exhibit better and faster acquisition in learning and memory processes, demonstrating a facilitation of spatial learning using the radial arm maze test (Braida et al., 2004). With regard to IL-1β, it is required for normal learning and memory processes, but exogenous administration or excessive endogenous levels may produce detrimental cognitive behavioral effects (Chen et al., 2008). A previous study demonstrated a synergistic interaction between IL-1β and other cytokines, causing enhanced cognitive dysfunction (Allan et al., 2005). Another recent study confirmed that IL-1β is involved in cognitive impairment after sepsis in rats (Mina et al., 2014), showing that IL-1β may be involved in brain dysfunction. TNF-α has been proven to play an important role in central nervous system development and functions including neuronal plasticity, cognition, and behavior (Garay and McAllister, 2010), where the disruption of TNF-α signaling leads to abnormal development of the hippocampus and impairments in cognitive function (Baune et al., 2012). TNF-α was initially described as a cell death inducer, and as a proinflammatory cytokine, it is generally recognized as a worsening factor in the pathology of psychiatric disorders. Several studies have suggested a link between increased TNF-α levels and cognitive alteration (Yirmiya and Goshen, 2011; Swardfager and Black, 2013), and a recent study showed that local increase of TNF-α in the hippocampal dentate gyrus activated astrocyte TNF receptor type 1 (TNFR1), which in turn triggered an astrocyte-neuron signaling cascade that resulted in persistent functional modification of hippocampal excitatory synapses (Habbas et al., 2015). On the other hand, TNF-α has also been suggested to have neuroprotective effects against cell death induced by various neurotoxic insults (Orellana et al., 2007; Rojo et al., 2008). Recent studies showed that decreased serum TNF-α level in chronic schizophrenia patients was significantly negatively correlated with psychopathological symptoms (Lv et al., 2015). Collectively, there is a positive association between IL-6 and IL-1β levels and impairment in brain function. On the other hand, TNF-α seems to have a more complex functionary mechanism, where it plays a dual role in producing either neurodegeneration or neuroprotection in the central nervous system (Orellana et al., 2007).

Ketamine has been shown to modulate the inflammatory response. Previous studies showed that ketamine had an anti-inflammatory effect under inflammation conditions and therefore recommended it for use in the surgery of sepsis patients (Takahashi et al., 2010; Ward et al., 2011). In a chronic stress-induced depression model, 10 mg/kg ketamine injection showed a rapid antidepressant effect and effectively reduced the protein expression levels of IL-6, IL-1β, and TNF-α (Wang et al., 2015). In a septic rat model, low dose ketamine (0.5 and 5 mg/kg) had an anti-inflammatory effect, but high dose ketamine (50 mg/kg) induced the expression of inflammatory cytokines (Sun et al., 2004). In primary cortical cultures, a sustained increase in IL-6 and a slight decrease in TNF-α were observed during ketamine (0.5 μM) exposure *in vitro* (Behrens et al., 2008). Another experiment *in vitro* found that in the presence of lipopolysaccharide (LPS), treatment with 100 μM ketamine for 30 min significantly increased IL-6 and TNF-α levels (Meng et al., 2015). These findings suggest a close relationship between ketamine and the inflammatory cytokines IL-6, IL-1β, and TNF-α; moreover, ketamine may have different regulatory effects on inflammatory cytokines under different conditions.

Our present study differed from previous investigations using the animal models induced by stress, LPS or sepsis, where we directly determined hippocampal cytosol protein levels of IL-6, IL-1β, and TNF-α by using acute and chronic ketamine administration models. We found that single ketamine administration increased the protein levels of IL-6 (only at the dose of 80 mg/kg), IL-1β, and TNF-α; multiple and long-term administration of ketamine increased the protein levels of IL-6 (only at 80 mg/kg when subjected to multiple injection) and IL-1β but significantly decreased the protein level of TNF-α in mouse hippocampus. These results indicate that the effects of ketamine on the levels of IL-6 and TNF-α are dose-dependent. Most importantly, the effects of ketamine administration on the level of TNF-α may be diametrically opposite under different conditions, depending on the dose and duration of administration. A sustained and repetitive stimulation could decrease the TNF-α level, whereas single administration produced different results. Our inference is well supported by a recent study in which the authors measured serum TNF-α, IL-6, and IL-18 levels in the 155 chronic ketamine abusers and 80 healthy control subjects, and observed increased protein expression levels of IL-6 and IL-18, whereas a significant decreased protein expression level of TNF-α (Fan et al., 2015). As we mentioned above, TNF-α may play a dual role in promoting either neurodegeneration or neuroprotection in the central nervous system; it seems that the effects of TNF-α on brain function may be inconsistent under different conditions. In chronic status, TNF-α may manifest beneficial rather than harmful effects and there may be a bi-directional regulatory pathway of TNF-α under different circumstances (Fan et al., 2015). The increased TNF-α level induced by single administration of ketamine facilitates neuroinflammation, but conversely, continuous repetitive stimulation by ketamine decreases TNF-α level, thereby weakening the neuroprotective effect of TNF-α and further aggravating the neurotoxicity of ketamine in the central nervous system. We also directly determined hippocampal mRNA levels of IL-6, IL-1β, and TNF-α, and most of results were consistent with the changes of corresponding protein levels. But to our surprise, the mRNA level of TNF-α after multiple ketamine administration was still significantly increased, which was contrary to the changing tendency of TNF-α protein level. This may be due to the increased protein degradation of TNF-α, although the exact reason is unclear.

The underlying molecular mechanism by which ketamine affects expression of IL-6, IL-1β, and TNF-α needs to be further explored. Toll-like receptor 4 (TLR4) has been suggested to be responsible for LPS recognition and subsequent production of pivotal inflammatory cytokines, such as IL-1β, IL-6, and TNF-α (Beutler and Rietschel, 2003). It has been shown that ketamine produces a proinflammatory effect by increasing TLR4 expression in a NMDA-independent manner. In a recent study, TLR4 was silenced with TLR4-siRNA vector and found that after TLR4-siRNA transfection, RAW264.7 cells pretreated with ketamine no longer promoted IL-6 and TNF-α expression in the presence of LPS (Meng et al., 2015). However, the exact mechanism of how ketamine induces dual function of TNF-α under different conditions still remains to be determined.

Although, the present study indicates that inflammatory cytokine levels may be involved in behavioral changes in mice, there are still limitations. After all, the spatial recognition memory and emotional response in mice did not strictly correspond to the alterations in the levels of IL-6, IL-1β, and TNF-α induced by ketamine, so some other factors must exist. Moreover, it is still unknown whether the behavioral performances would change if the expression of these inflammatory cytokines were silenced. In future studies, we should focus on the underlying mechanism, exploring the exact roles of IL-6, IL-1β, and TNF-α in spatial memory deficits and emotional response.

## Conclusion

We found that chronic ketamine administration at the dose of 60 mg/kg (not 30 mg/kg) induced spatial recognition memory deficit and reduced anxiety-like behaviors in mice. Moreover, our findings indicate that ketamine can increase the levels of the inflammatory cytokines IL-6 and IL-1β, which would lead to neuroinflammation. Interestingly, the effects of ketamine on the level of TNF-α are inconsistent under different conditions, depending on the dose and duration. Single ketamine administration can increase TNF-α level, while sustained and repetitive stimulation decrease it. These results reveal new insights that alterations in the levels of IL-6, IL-1β, and TNF-α may play an important role in the neurotoxicity of ketamine.

## Author Contributions

XW, YLu, and YLi conceived and designed the experiments. YLi, RS, GW, RD, AD, and ZD performed the experiments. JZ and GW helped to analyze and interpret the data. YLi and RS drafted the manuscript. XW, YLu, RZ, and GZ provided critical revision. All authors reviewed and approved the final manuscript.

## Conflict of Interest Statement

The authors declare that the research was conducted in the absence of any commercial or financial relationships that could be construed as a potential conflict of interest.
